# Prevalence of suicidal behavior and its associated factors among individuals living in war-affected areas of Dessie Town, northeast Ethiopia, in 2022: a cross-sectional study

**DOI:** 10.3389/fpsyt.2025.1453526

**Published:** 2025-05-29

**Authors:** Zelalem Birhan, Maregu Shegaw, Kunuya Kunno, Tamrat Anbesaw, Lebasie Woretaw, Assasahegn Tedla, Birhanu Mengist Munie, Asmare Belete

**Affiliations:** ^1^ Department of Psychiatry, College of Medicine and Health Science, Wollo University, Dessie, Ethiopia; ^2^ Department of Environmental Health Science, College of Medicine and Health Science, Wollo University, Dessie, Ethiopia; ^3^ Department of Psychiatry, College of Health Science, Debre Tabor University, Debre Tabor, Ethiopia

**Keywords:** communities, Ethiopia, suicide prevention, war-affected areas, war

## Abstract

**Background:**

Globally, suicide is a serious public health issue, especially in areas devastated by conflict where trauma and psychological anguish are prevalent. War has long-lasting impacts on mental health, particularly increasing the risk of suicidal behavior among survivors. Suicidal ideas and attempts must be promptly identified and handled, even though suicide cannot be directly treated. The prevalence of suicidal behavior in war-affected areas remains poorly understood. Existing studies often focus on high-income countries, leaving a gap in knowledge for low- and middle-income countries like Ethiopia, specifically in Dessie Town. This study aimed to assess the prevalence of suicidal behavior and its associated factors among individuals living in a war-affected area, Dessie Town, Northeast Ethiopia.

**Methods:**

A community-based cross-sectional study was conducted among adult residents in the war-affected area, Dessie Town. A total of 617 individuals were selected by a systematic random sampling method. Suicidal behavior was assessed using the Mini International Neuropsychiatric Interview suicidal behavior screening tool. Multivariable logistic regressions were used to measure the associated factors. Associations of variables were described by using odds ratios (ORs), 95% confidence intervals (CIs), and p-values less than 0.05.

**Results:**

The prevalence of suicidal behavior in these participants was 15.3% (95% CI: 12.5, 18.3). Factors significantly associated with suicidal behavior included being divorced or widowed (AOR: 2.19, CI: 1.06, 4.50), being unemployed (AOR: 2.11, CI: 1.16, 3.83), having depression (AOR: 3.11, CI: 1.80, 5.39), having post-traumatic stress disorder (PTSD) (AOR: 1.96, CI: 1.12, 3.43), stressful life events (AOR: 3.80, CI: 1.97, 7.36), and having poor social support (AOR: 3.40, CI: 1.71, 6.78).

**Conclusion and Recommendation:**

The findings highlight a significant burden of suicidal behavior among survivors of conflict-affected areas in northeast Ethiopia, an under-researched population, emphasizing the need for integrated mental health and psychosocial support services tailored to war-affected communities. The following are the determinants of suicide behavior: being divorced or widowed, unemployed, depressed, suffering from PTSD, experiencing stressful life events, and having inadequate social support. These groups should be prioritized for interventions in resource-limited settings. To improve and lessen the signs of suicidal behavior and to optimize the quality of life for those who survive post-war, intervention is necessary.

## Background

Armed conflict has profound and lasting effects on individuals’ mental health, particularly among those directly exposed to violence, displacement, and the loss of loved ones. One of the most concerning consequences of such traumatic experiences is the increased risk of suicidal behavior. Suicidal behavior encompasses a range of self-destructive behaviors, including suicidal ideation, planning, attempts, and death by suicide (1, 2). In regions devastated by conflict, suicidal behavior poses a serious threat to public health. When compared to non-affected areas, these regions have a higher rate of suicide ideation and attempts (3).

Suicide is the fourth most common cause of death for young individuals aged 15 to 29 for both sexes, and over half of all suicides worldwide (58%) happen before the age of 50 years (1). The global suicide death toll was predicted to reach 703,000 in 2019, with 77% of the deaths occurring in low- and middle-income countries (LMICs) (1). Furthermore, approximately 803,900 suicides occurred in 2012, accounting for 1.4% of the world’s illness burden or more than 39 million disability-adjusted life years (DALYs) lost, according to the Global Burden of Disease Report (4). Suicidal behavior is a complex and multifaceted issue, influenced by a vast range of interrelated factors across various levels of society. These include genetic, psychological, psychiatric, social, economic, and cultural risk factors, all of which interact within societal, community, interpersonal, and individual domains. The intricate interplay of these factors makes understanding and addressing suicidal behavior particularly challenging, especially in populations exposed to conflict and trauma (5).

Suicide has been one of the leading causes of death in the West, with the World Health Organization (WHO) predicting that 10 to 20 times as many individuals would attempt suicide worldwide in 2020, and that 1.53 million people would lose their lives to suicide (6). According to a systematic review of research on suicidal behavior in displaced individuals from 2022, the prevalence of suicidal ideation was 70.6% overall (7). The suicide rate among adult war-affected populations in eastern Uganda was 9.2% (8) and it was 16.29% (9) among high school students in the war-affected town of Woldia. Furthermore, the rates of suicidal ideation and attempt in war-affected internally displaced people in northwest Ethiopia were 22.4% and 6.7% (10), respectively. Thus, 75% of all suicides occur in LMICs, where limited resources are available to prevent suicidal behavior (11) due to a lack of mental health services and resources in these areas, resulting in devastation for individuals, families, and communities, and this can lead to long-term psychological trauma (3).

Research conducted in war-torn areas of sub-Saharan Africa, the Middle East, and Southeast Asia has consistently shown higher rates of suicidal behavior among survivors. This increase is often associated with experiences of trauma, displacement, and persistent socioeconomic challenges (12, 13). After experiencing trauma, particularly through war, the survivors often experience higher rates of depression, post-traumatic stress disorder (PTSD), and suicidal behavior, highlighting the enduring psychological effects of conflict (14). As a result, civilians who have been exposed to prolonged periods of warfare report increased instances of suicidal thoughts and attempts compared to the general population (15).

Millions of individuals and whole communities worldwide have suffered and continue to suffer from prolonged war-related traumatic stressors, such as high levels of distress amongst civilian survivors of war (16), which exacerbate existing mental health challenges and trauma. Moreover, displacement, loss, and disruption caused by armed conflicts contribute to psychological distress and lead to increased rates of suicidal behavior (17). However, LMICs face challenges due to limited resources and inadequate data for suicide prevention (18).

Factors such as trauma exposure; separation from one’s parents, lover, spouse, or friends, displacement; loss of loved ones; disrupted social support networks; exposure to violence, depression; post-traumatic stress disorder; substance abuse; poor social support; and loss of family members are associated with suicidal behavior in war-affected areas (19–23).

Ethiopia has faced numerous conflicts over the past few decades, including the Ethiopian Civil War in the late 20th century and more recent struggles such as the Tigray War. These conflicts have resulted in significant displacement, loss of life, and psychological trauma for many affected individuals (24). The Tigray conflict, which started in November 2020, has particularly devastated the northern regions of Ethiopia, including the Amhara region, where the city of Dessie is situated. Ethiopia’s official media have been reporting since July 2021 that there have been war attacks and other forms of mistreatment in cities and communities in northern Amhara, notably Dessie. Over 550,000 people had been displaced by mid-September 2021 as a result of the conflict, and hundreds of civilians had been slaughtered or killed by bombardment. Targeted killings; material destruction; the destruction of villagers’ homes, fields, cattle, and food supplies; and sexual abuse, have resulted in hundreds of thousands of internally displaced people (IDPs) (25).

In Ethiopia, the intricate socio-political environment, influenced by historical conflicts and recent unrest, has heightened concerns about mental health problems, such as suicidal behavior, in communities affected by war. The country has endured various conflicts, including a prolonged civil war and more recent regional skirmishes, which have thus led to considerable mental health difficulties, especially among displaced individuals and those who have survived violence (26). While there is substantial global evidence connecting war exposure to an increased risk of suicide, the specific factors that contribute to suicidal behavior in this area, such as cultural stigma, social disruption, and the availability of mental health services, are not well understood. There is an urgent need for localized research, especially since Ethiopia has experienced prolonged ethnic and political conflicts, particularly in its northern regions (27).

Dessie, a city with deep historical roots in the Amhara region, has been greatly affected by the ongoing conflict. Its strategic position has turned it into both a battleground and a safe haven for IDPs escaping violence from surrounding areas. The city is grappling with numerous challenges, such as damaged infrastructure, economic turmoil, and an increasing strain on healthcare services. The psychological impact of war, along with displacement and a lack of resources, has led to a rise in mental health problems, including an increased risk of suicide, among both residents and displaced individuals in the area.

However, there is a lack of empirical studies that specifically examine suicidal behavior among survivors of conflict in northeast Ethiopia. One study carried out in the general population indicated that suicidal thoughts were common among individuals facing mental health issues, but it did not concentrate specifically on those who survived war (28).

This study seeks to examine the prevalence of suicidal behavior among survivors in northeast Ethiopia and to investigate its connections with various sociodemographic and psychological factors. These factors encompass trauma history, mental health disorders (including depression and PTSD), and socioeconomic status. The selected variables are grounded in existing research that underscores the intricate relationship between conflict exposure, mental health, and suicidal behavior.

This study holds significant importance as it is among the first to concentrate on war survivors in northeast Ethiopia, offering essential insights into the mental health issues encountered by this marginalized group. The results will enhance the current understanding of suicide prevention in areas affected by conflict and will help shape public health initiatives in Ethiopia and comparable settings. By exploring the sociodemographic and psychological factors that affect suicidal behavior, this research aims to provide evidence that can inform customized mental health services for war survivors.

## Methods

### Study area and study period

The investigation was carried out from 21 June to 21 July 2022, in Dessie Town, South Wollo Zone, northeast Ethiopia. Ethiopia’s official media have been reporting since July 2021 that there have been war attacks and other forms of mistreatment in cities and communities in northern Amhara, notably Dessie. Over 550,000 people had been displaced by mid-September 2021 as a result of the conflict, and hundreds of civilians had been slaughtered or killed by bombardment. Targeted killings; material destruction; the destruction of villagers’ homes, fields, cattle, and food supplies; and sexual abuse have resulted in hundreds of thousands of IDPs (25). Addis Ababa is 401 kilometers away from Dessie Town, while Bahir Dar is 490 kilometers away. There are five sub-cities in the town, with 8 rural and 18 urban kebeles. The 2022 report from the Dessie city government states that of the population of 285,530 of Dessie Town, approximately 244,724 reside in urban areas and 40,806 reside in rural areas.

### Study design

A community-based cross-sectional study was conducted.

### Population

The source population consisted of all adult Dessie Town residents, while the study population consisted of adult Dessie Town residents living in selected households during the study period.

### Eligibility criteria

Inclusion criteria: The study only included residents who were above the age of 18 and had been in Dessie Town for more than 6 months.

Exclusion criteria: Those who were unable to give proper information, such as those who were unconscious, seriously ill, or unable to communicate, were excluded from the study.

### Sample size determination

The sample size for this investigation was established using a single population proportion formula, which was based on the estimated 58.3% prevalence rate of suicidal behavior in South West Ethiopia (29). With a 95% confidence level, a 5% margin of error, and a 10% non-response rate, after applying the formula, the sample size was 411. Therefore, after applying a design effect of 1.5, the final sample size was 617.

### Sampling procedure

**Figure 1:**
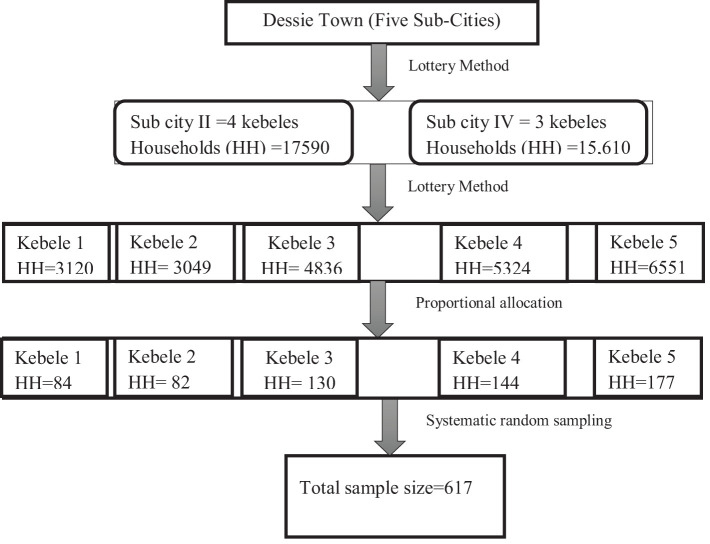
Schematic presentation of the sampling procedure for this study on the prevalence of suicidal behavior and associated factors among individuals living in a war-affected area in Dessie Town, northeast Ethiopia, in 2022.

The sampling method was a multi-stage systematic random sampling technique. The lottery method was used to choose two sub-cities at random from a total of five sub-cities. From a chosen sub-city, five kebeles were chosen by lottery, and the sample size was allocated to each kebele in proportion to the number of households. After choosing a starting household through a lottery, households in the chosen kebele were chosen using a systematic random sampling procedure (**Figure 1**). Interviews were conducted with eligible members of the chosen family. When there were multiple eligible participants in the home, only one was included using the lottery procedure. The household was recorded as non-response if the interviewer was unable to locate an eligible participant at a specific time. The interviewer returned to the household three times at different intervals. The next household was used after that.

### Data collection tools and procedures

From the psychiatry field, five BSc data collectors and two MSc supervisors were chosen, and they received instruction from the primary investigator regarding data gathering techniques and resources. A pretest was administered to 31 people outside the study area in Kombolcha Town 2 weeks before the actual data collection took place to ensure that the questionnaires were clear. All the tools were carefully selected for their validity and applicability in the Ethiopian context. Psychiatric specialists translated a questionnaire into Amharic, using a forward-backward translation procedure, and then a third party translated it back into English to ensure consistency and tool understandability. The lead investigator and the supervisors conducted routine oversight to guarantee that all the required data were appropriately gathered. Before the data were processed and transferred from paper to a computer, it was cleaned and verified to be complete.

Six sub-sections of a structured interviewer-administered questionnaire were used:

Suicidal behavior: Being at risk for suicidal behavior was assessed using a suicidal screening tool, which is part of the Mini International Neuropsychiatric Interview (MINI), and it was set based on the Diagnostic and Statistical Manual of Mental Disorders-IV criteria. It consists of six items that are scored “yes or no”. Among these items, 1–5 record whether an event has occurred during the last month, and item 6 records the lifetime occurrence of the event. Participants are deemed at risk for suicidal behavior if they answer “yes” to any one of the six items in the MINI suicidal screening tool (30). This tool is short and structured and has good validity and reliability in screening those deemed at risk for suicidal behavior. The internal consistency of the six MINI items was 0.84 (31). It has been utilized in Ethiopia (32).

The 17 DSM-IV symptoms of PTSD were evaluated using the Post-Traumatic Stress Disorder Civilian Version (PCL-C), a self-report rating scale that is simple to administer. With a cut point of ≥50 (33), the total score is calculated by summing the 17 elements, allowing possible scores to range from 17 to 85 using a five-point Likert scale (1 = Not at all, 2 = A little bit, 3 = Moderately, 4 = Quite a bit, and 5 = Extremely). This instrument was adapted from a study conducted among Somali and Oromo Ethiopians in Minnesota (34). It has a sensitivity of 0.778 and a specificity of 0.86 (35). An internal consistency value of above 0.75 was found in an adult community (36). It has been utilized in Ethiopia (37).

The Patient Health Questionnaire-9 (PHQ-9) measures depression if a person scores 10 or above on at least nine of its items. It has four-scale responses consisting of not at all (0), several days (1), more than half the days (2), and nearly every day (3) over the last 2 weeks (38). It has been validated in Ethiopia to screen for major depressive disorder among adults in Ethiopia. It showed a good internal Cronbach’s alpha value of 0.81, with a sensitivity of 86% and specificity of 67% (38).

Utilizing the Oslo Social Support Scale (OSS-3), the social support of the patients was evaluated. The cumulative score scale for the OSS-3 ranges from 3 to 14. OSS-3 scores of 3–8 indicate poor social support, 9–11 indicate moderate social support, and 12–14 indicate strong social support (39). The List of Threatening Experiences (LTE) questionnaire was used to rate stressful experiences with yes/no responses. Participants reporting ≥1 event from the 12-item list were considered to have experienced a major life stressor. The LTE comprises 12 distinct items representing stressful events and demonstrated strong test-retest reliability (alpha coefficient of 0.74). It is a legitimate and trustworthy indicator of stress in mental health (40), and it has been utilized in Ethiopia (37).

Additionally, to screen for problematic substance use (alcohol, khat, tobacco, and cannabis), the Cut down, Annoyed, Guilty, and Eye-opener- Adapted to Include Drugs (CAGE-AID) questionnaire was utilized. For the CAGE questions, “no” answers received a score of 0, and “yes” answers received a score of 1. Problematic substance use was defined as those who provided two or more positive responses (41).

A sociodemographic questionnaire was used to assess the patients’ background information. Clinical and trauma factors were assessed by yes/no answers from the respondents and were operationalized according to different studies.

### Data processing and analysis

The completeness of the data was checked before coding and entry into EpiData version 4.6, and then exported to SPSS version 25 for analysis. Descriptive statistics were presented using frequency tables, charts, and figures. Bivariable logistic regression was conducted to identify candidate variables for multivariable analysis. Variables with a p-value ≤ 0.25 in the bivariable analysis were included in the multivariable logistic regression model to control for potential confounders. Adjusted odds ratios (AORs) with 95% confidence intervals (CIs) were used to assess the strength and direction of associations. A p-value < 0.05 was considered statistically significant. Model fitness was assessed using the Hosmer and Lemeshow goodness-of-fit test.

## Results

### Sociodemographic characteristics of the respondents

A total of 600 participants participated, with a response rate of 97.2. The mean age of the respondents was 39.87 years with an SD ±10.063 years, and most respondents (303 or 50.5%) were between the ages of 31 and 45 years. The majority of the respondents were male (374 or 62.3%), married (439 or 73.2%), had a diploma or higher (230 or 38.3%), had a job (389 or 64.8%), and had a low income (325, or 54.2%) (**Table 1**).

**Table 1:** Sociodemographic characteristics of study participants among individuals living in a war-affected area in Dessie Town, northeast Ethiopia, in 2022 (n=600).

Characteristic	Category	Frequency	Percentage
Age	18–30	133	22.2
	31–45	303	50.5
	>45	164	27.3
Sex	Female	226	37.7
	Male	374	62.3
Marital status	Married	439	73.2
	Single	84	14.0
	Divorced/widowed	77	12.8
Educational status	No formal education	60	10.0
	Primary schooling	209	34.8
	Secondary schooling	101	16.8
	Diploma and above	230	38.3
Occupational status	Unemployed Employed	211 389	35.2 64.8
Income (in ETB)	Low income	325	54.2
	Medium income	204	34.0
	High income	71	11.8

ETB, Ethiopian Birr.

### Clinical and psychosocial factors

Out of all the participants, 44 (7.3%) had a family history of documented mental illness, and 63 (10.5%) had a diagnosed chronic medical condition. When asked if they were currently depressed, 192 (32% of survey participants) said they were. In this study, the prevalence of PTSD was 34.5% and problematic substance use was 19.8%. Of the participants, 276 (46%) had suffered a stressful life event within the 6 months after the start of the conflict, 120 (20%) had been forced to separate from their families during the crisis, and 264 people (44%) had strong social support (**Table 2**).

**Table 2:** Clinical and psychosocial factors of the respondents living in a war-affected area in Dessie Town, northeast Ethiopia, in 2022 (n=600).

Clinical factor	Category	Frequency	Percentage
Family history of diagnosed mental illness	Yes	44	7.3
	No	556	92.7
Diagnosed with a chronic medical condition	Yes	63	10.5
	No	537	89.5
Current depression status	Yes	192	32.0
	No	408	68.0
Post-traumatic stress disorder	Yes	207	34.5
	No	393	65.5
Problematic substance use	Yes	119	19.8
	No	481	80.2
Property destroyed during the disaster	Yes	138	23.0
	No	462	77.0
Sexually abused or raped	Yes	29	4.8
	No	571	95.2
Experienced forced separation from family	Yes	120	20.0
	No	480	80.0
Stressful life event	Yes	276	46.0
	No	324	54.0
Social support	Poor	191	31.8
	Intermediate	145	24.2
	Strong	264	44.0

### Prevalence of suicidal behavior

Of the respondents, 56 (9.3%) said they had wished they were dead in the previous month, 42 (7%), 38 (6.3%) said they had wanted to commit suicide in the previous month, 25 (4.2%) had planned how to commit suicide in the previous month, 23 (3.8%) said they had attempted suicide in the previous month, and 33 (5.5%) had ever tried suicide (**Table 3**).

**Table 3:** The prevalence of suicidal behavior among individuals living in a war-affected area in Dessie Town, northeast Ethiopia, in 2022. (n=600).

Variable	Category	Frequency	Percentage
Wished you were dead in the previous month	No	544	90.7
	Yes	56	9.3
Wanted to harm yourself in the previous month	No	558	93.0
	Yes	42	7.0
Thought of committing suicide over the previous month	No	562	93.7
	Yes	38	6.3
Planned how to commit suicide in the previous month	No	575	95.8
	Yes	25	4.2
Attempted suicide in the previous month	No	577	96.2
	Yes	23	3.8
Ever attempted suicide	No	567	94.5
	Yes	33	5.5

**Figure 2:**
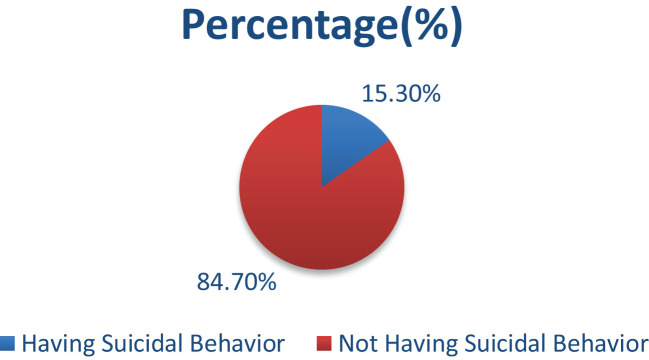
The prevalence of suicidal behavior among individuals living in a war-affected area in Dessie Town, northeast Ethiopia, in 2022.

The overall prevalence of suicidal behavior in these participants was 15.3% (95% CI: 12.5, 18.3) (**Figure 2**).

#### Factors associated with suicidal behavior

In the multivariable analysis, being divorced or widowed (AOR: 2.19, CI: 1.06, 4.50), being unemployed (AOR: 2.11, CI: 1.16, 3.83), having depression (AOR: 3.11, CI: 1.80, 5.39), having PTSD (AOR: 1.96, CI: 1.12, 3.43), stressful life events (AOR: 3.80, CI: 1.97, 7.36), and poor social support (AOR: 3.40, CI: 1.71, 6.78) were found to be significantly associated with suicidal behavior (**Table 4**).

**Table 4:** Bivariable and multivariable independent factors for suicidal behavior among individuals living in a war-affected area in Dessie Town, northeast Ethiopia, in 2022 (n= 600).

Variable	Category	Suicidal behavior		COR (95%CI)	AOR (95%CI)
		Yes	No		
Marital status	Married	56	383	1	1
	Single	17	67	1.74 (0.95, 3.17)	1.23 (0.57, 2.72)
	Divorced/ Widowed	19	58	2.24 (1.24, 4.04)	2.19 (1.06, 4.50)*
Age	18–30	29	104	1	1
	31–45	40	263	0.55 (0.32, 0.93)	0.67 (0.34, 1.33)
	>45	23	141	0.59 (0.32. 1.07)	0.85 (0.36, 2.01)
Occupation	Employed	56	333	1	1
	Unemployed	36	175	1.22 (0.78, 1.93)	2.11 (1.16, 3.83)*
Educational status	Illiterate	9	51	0.81 (0.37.1.78)	1.40 (0.46, 4.22)
	Grades 1–8	27	182	0.68 (0.40, 1.16)	0.78 (0.39, 1.55)
	Grades 9–12	15	86	0.80 (0.42, 1.53)	0.86 (0.40, 1.85)
	Diploma and above	41	189	1	1
Depression	Yes	59	133	5.04 (3.15, 8.06)	3.11(1.80, 5.39)**
	No	33	375	1	1
PTSD	Yes	49	158	2.52 (1.61, 3.96)	1.96 (1.12, 3.43)*
	No	43	350	1	1
Property destroyed during the disaster	Yes	40	98	3.22 (2.02, 5.14)	1.26 (0.68, 2.33)
	No	52	410	1	1
Forced separation from family	Yes No	32	88	2.55 (1.57, 4.14)	1.10 (0.59, 2.06)
		60	420	1	1
Stressful life event	Yes	76	200	7.32 (4.15, 12.91)	3.80 (1.97, 7.36)**
	No	16	308	1	1
Social support	Poor	53	138	4.95 (2.82, 8.71)	3.40 (1.71, 6.78)**
	Intermediate	20	125	2.06 (1.06, 4.01)	1.53 (0.69, 3.38)
	Strong	19	245	1	1

1, reference group; *p<0.05; **p<0.01; COR, crude odds ratio; AO, adjusted odds ratio.

## Discussion

Suicidal behavior is a significant public health concern in war-affected areas, with debilitating consequences for societal, community, and individual relationships. This study showed that the prevalence of suicidal behavior among the participants was 15.3% (95% CI: 12.5, 18.3) in the war-affected area of Dessie Town. This result was in line with other findings that reported a 14.6% prevalence of suicidal behavior amongst school-going adolescents in post-conflict Sierra Leone (42); 16.29% and 12.87% (9), respectively, for suicidal ideation and attempts among high school students in Woldia Town, Ethiopia; and 16.2% for suicide attempts among high school students in Dangila Town, Ethiopia (43).

However, our finding was lower than in other studies, including 40% for suicidal behavior after trauma in Sweden (44), suicidality prevalence of 38.3% among civilian residents who experienced trauma (19), 30.3% of asylum-seekers reported having experienced suicidal ideation in Germany (45), 19% war-exposed civilian population of Nepal reported any lifetime suicidal ideation (46), 27.5% of survivors attempted suicide in the United States (47), and the prevalence of suicidal behavior after severe trauma in Sweden (40% had attempted suicide, 29% had a detailed suicide plan, and 31% had recurrent suicidal thoughts) (48). This disparity could be attributed to the presence of comorbid conditions such as PTSD and depression, which would increase suicidal thoughts. Thus, different studies indicated that the rate of suicidal behavior increased as comorbid PTSD and depression increased (19, 44).

Another reason for the variation could be that the presence of a long-lasting war and severe trauma during the disaster could lead to a greater tendency to have suicidal tendencies, as shown in different studies (46), while the war lasted for 6 months in our study. Moreover, the discrepancy may be due to cultural variations across different nations. Another possible reason for the discrepancy may be a lack of knowledge and attitude toward suicidal behavior, as participants may hide their suicidal behavior. The Ethiopian community’s cultural and religious beliefs often stigmatize suicide, viewing it as morally or religiously unacceptable. Such stigma may discourage individuals from acting on suicidal thoughts or reduce the likelihood of suicide being reported, which may contribute to lower reported rates (49, 50).

The relatively low prevalence observed in our study could be due to protective factors common in northeast Ethiopia, including religion, family support, and community cohesion, which have been found to help reduce suicidal thoughts. Additionally, faith-based coping strategies and strong community connections are widespread in the region and have consistently been associated with a lower risk of suicide in similar cultural settings (51, 52). These variations highlight the complex factors that contribute to suicide risk, such as the length and intensity of displacement, availability of mental health services, and experiences of violence. Conversely, the participants in our study, despite being impacted by war, may have relatively stronger community support systems, which could have reduced the overall risk.

In addition, our finding was lower than in other studies conducted in northwest Ethiopia, which revealed that the suicidal ideation prevalence among war-affected IDPs was 22.4% (10). A possible reason for this discrepancy could be due to suicide rates among IDPs differing greatly and, in some situations, may exceed those of the general population. A higher suicide rate among IDPs can be linked to various factors, such as exposure to conflict, loss of livelihood, separation from family, and limited access to mental health services. These challenges lead to a greater occurrence of mental health issues, including depression and posttraumatic stress disorder, which are connected to a heightened risk of suicide (7).

However, our result was higher than that in other studies, including a prevalence of 9.2% of lifetime attempted suicidal behavior in the adult war-affected resident population of eastern Uganda (8), 11.6% for past experience of suicide in Uganda (53), 6.13% of the population experienced current suicidal ideation in a rural population of adults in Haiti’s Central Plateau (54), 7.1% of respondents reported that they had suicidal ideation at some point in their lives in the general population of Afghanistan after traumatic event experiences (15), and 6.1% for suicidal behavior in residents of rural northeastern Uganda (55). This disparity may be due to the fact that our study focused on participants who experienced traumatic events during the war, whereas other studies have studied the general population without experiencing traumatic events, like those in Uganda (55). Another explanation could be that this study was conducted shortly after the conflict finished, which implies that performing research after a war ends raises the possibility that recall bias will cause the prevalence to be lower. The population in northeast Ethiopia has endured extended and intense trauma from ongoing conflict, which may have led to increased psychological distress. Furthermore, differences in methodology, such as employing more sensitive assessment tools and broader definitions of suicidal behavior in our research, encompassing not only ideation but also past attempts or other related risk factors, could have led to a higher prevalence estimate and could explain the higher prevalence observed. The increased displacement and lack of resources in northeast Ethiopia could have further heightened the risk (10). Compared to populations in Uganda or Afghanistan, the participants in our study may have experienced more severe or recent trauma, exacerbating their psychological distress.

In this study, the odds of suicidal behavior were 2.19 times higher in those divorced/separated than in married respondents. The possible explanation is that loneliness is typically associated with the subjective feeling of social isolation, thus resulting in reduced social skills, maladaptive ways of thinking, and feelings that are indirectly related to suicidal ideation and attempts (56, 57). The extra burden of bereavement and grief may deepen in situations where the conflict hinders normal support structures. A bereavement can negatively impact one’s mental health by triggering a depressive state, an anxious state, or even escalating suicidal tendencies. In other cases, the majority of the emotional stresses surrounding grief and loss may be worsened by the conflict stressors in the area where the grieving person lives (58). Participants who were unemployed were 2.11 times more likely to develop suicidal behavior compared with those who were employed. A possible reason may be a loss of self-esteem, a sense of isolation among individuals, and reluctance to communicate the family members due to fear of societal judgments, which thus results in suicidal thoughts (59).

In addition to these financial burdens, unemployment often translates into a lack of purpose, reduced independence, and economic insecurity, all of which are important for mental health. Furthermore, the absence of employment can destroy the future hopes of an individual, leaving them feeling confined and powerless. These factors together serve to increase their vulnerability to poor mental health outcomes (60). The odds of developing suicidal behavior were 3.11 times higher among individuals who had depression when compared to respondents without depression. This could be because participants with depression are more likely to have a diathesis for suicidal behavior. There are complex interactions between genes and the environment that raise the risk of neurobiological alterations (abnormalities in the hypothalamic adrenal axis and the noradrenergic and serotonergic systems in suicidal behavior and major depression) (61). Persistent feelings of sadness and hopelessness and a loss of interest in daily activities can severely impair an individual’s ability to cope with the trauma of war (60). This is supported by previous studies of suicide in war-affected areas of Uganda (8).

Those who had PTSD were 1.96 times more likely to have suicidal behavior than those who did not. This could be the result of recurrent or long-lasting childhood trauma, such as physical or sexual abuse, which has been connected to the emergence of personality disorders, PTSD, impulsivity, and violence. Suicidality risk is elevated in individuals with PTSD and a disordered pre-trauma psychiatric state due to their coping strategies (62). This is consistent with previous studies of suicidal behavior in Germany (45) and the war-affected area of Woldia, Ethiopia (9).

Participants who had experienced a stressful life event were 3.80 times more likely to have suicidal behavior than those who had not experienced an event. This may be due to stressful life events increasing suicidality by increasing psychological distress and eroding perceived social support, resulting in passive coping styles, and thus increasing suicidal behavior (63). The likelihood of developing suicidal behavior was 3.40 times higher among respondents who had poor social support when compared to respondents who had good social support. Poor social support from friends and family may be the cause of this, which can lead to feelings of loneliness and a decrease in social connectivity without the means to handle stress, which can heighten the risk of suicidal thoughts and actions (64). Strong social connections can help protect against psychological distress and suicidal thoughts, particularly during adversity (65). This is consistent with a previous study in Dangila Town, Ethiopia (43). To address this issue, public health initiatives should focus on enhancing social support networks, especially for at-risk groups such as displaced individuals and survivors of conflict. Community programs, peer support groups, and family counseling are essential in building resilience and lowering the risk of suicide. Furthermore, targeted awareness campaigns can help diminish stigma and promote the creation of supportive environments.

Interestingly, our study did not reveal a significant link between age and suicide risk, which contrasts with findings in other groups where younger or older individuals are typically seen as more at risk. This may be due to the uniform exposure to trauma within our study population, where the common experience of conflict could have overshadowed age-related differences. Cultural norms in northeast Ethiopia, where older individuals are held in high regard and younger people benefit from strong family connections, may play a role in reducing age-related suicide risks. In addition, our study found no significant link between educational status and suicide risk, which contrasts with other studies where lower education and younger age are often linked to higher suicide risk (66). This discrepancy may be due to the profound effects of trauma and displacement, which can impact individuals regardless of their educational background. Furthermore, in rural and conflict-affected areas, having a higher education level may not offer substantial socioeconomic benefits, thereby diminishing its protective influence. These results highlight the importance of targeted interventions that focus on trauma-related stressors while also harnessing community resilience.

These findings indicate that in populations affected by conflict, trauma and displacement are the main contributors to suicide risk, and interventions should focus on addressing these issues. Additionally, it is essential to understand the impact of cultural and community coping mechanisms to create effective prevention strategies. This study emphasizes the pressing need for public policies that prioritize mental health and social support for vulnerable groups, especially those impacted by war and displacement. The significant rate of suicide risk found among the study participants highlights the importance of incorporating mental health services into Ethiopia’s primary healthcare framework. Policies should focus on increasing access to mental healthcare, training healthcare professionals, and ensuring facilities are equipped to effectively address mental health issues.

Moreover, social assistance initiatives aimed at displaced individuals and survivors of conflict are essential for tackling the social factors that influence mental health. These initiatives could involve offering psychosocial support, alleviating economic difficulties, and developing community-based programs to build resilience. Implementing these strategies is crucial for reducing the long-term psychological and social effects of war on those affected. We emphasize the need for mental health and social assistance measures, noting that our findings indicate the necessity for specialized interventions targeting at-risk persons in war-affected communities.

### Limitations of the study

One limitation of this study is selection bias. While systematic sampling was employed, it is possible that vulnerable populations, such as individuals with severe mental health issues or those in extreme isolation, were left out. These groups are likely at a higher risk for suicide, which could lead to an underestimation of the actual prevalence of suicidal behavior. Recall bias may be explained by the fact that people who do not engage in suicidal behavior may be less motivated than people who do remember past exposures. The participants may have a tendency to give socially acceptable answers to delicate questions about sexual abuse and problematic substance use, which could lead to social desirability bias.

### Conclusion

The findings highlight a significant burden of suicidal behavior among survivors in conflict-affected areas in northeast Ethiopia, an under-researched population, emphasizing the need for integrated mental health and psychosocial support services tailored to war-affected communities. The following are the determinants of suicidal behavior: being divorced or widowed, unemployed, depressed, suffering from PTSD, experiencing stressful life events, and having inadequate social support. These groups should be prioritized for interventions in resource-limited settings. In order to improve and lessen the signs of suicidal behavior and to optimize the quality of life for those who survive after war, intervention is necessary.

## Data Availability

The raw data supporting the conclusions of this article will be made available by the authors, without undue reservation.

## References

[1] World Health Organization (WHO). Suicide worldwide in 2019: global health estimates. (2021).

[2] Joiner T. Why people die by suicide. Cambridge, Massachusetts, USA: Harvard University Press (2005).

[3] Jankovic J Bremner S Bogic M Lecic-Tosevski D Ajdukovic D Franciskovic T. Trauma and suicidality in war affected communities. Eur Psychiatry. (2013) 28:514–20. doi: 10.1016/j.eurpsy.2012.06.001 22986125

[4] Sharmin Salam S Alonge O Islam MI Hoque DME Wadhwaniya S Ul Baset MK. The burden of suicide in rural Bangladesh: Magnitude and risk factors. Int J Environ Res Public Health. (2017) 14:1032. doi: 10.3390/ijerph14091032 28891939 PMC5615569

[5] Platt S Arensman E Rezaeian M. National suicide prevention strategies–progress and challenges. Gottingen, Germany: Hogrefe Publishing (2019). 10.1027/0227-5910/a000587 30892084

[6] Gvion Y Apter A. Suicide and suicidal behavior. Public Health Rev. (2012) 34:1–20. doi: 10.1007/BF03391677 26236074

[7] Cogo E Murray M Villanueva G Hamel C Garner P Senior SL. Suicide rates and suicidal behaviour in displaced people: A systematic review. PloS One. (2022) 17:e0263797. doi: 10.1371/journal.pone.0263797 35271568 PMC8912254

[8] Kinyanda E Weiss HA Mungherera M Onyango-Mangen P Ngabirano E Kajungu R. Prevalence and risk factors of attempted suicide in adult war-affected population of eastern Uganda. Crisis. (2013) 34:367–74. doi: 10.1027/0227-5910/a000196 23608229

[9] Kassa MA Srahbzu M Nenko G Nakie G Mekuria K Feleke SF. Suicidal ideation and attempts among high school students of war-affected area at Woldia town, Northeast, Ethiopia, 2022. BMC Psychiatry. (2023) 23:384. doi: 10.1186/s12888-023-04889-4 37259028 PMC10234009

[10] Tadesse G Gashaw F Zeleke TA Fentahun S Yitayih S. Prevalence and factors associated with suicidal ideation and attempts among war-affected internally displaced people in northwest Ethiopia, 2022. BJPsych Open. (2024) 10:e132. doi: 10.1192/bjo.2024.71 39086297 PMC11698158

[11] Organization WH. Preventing suicide: A global imperative. Geneva, Switzerland: World Health Organization (2014).

[12] Fleischmann A De Leo D. The World Health Organization’s report on suicide: a fundamental step in worldwide suicide prevention. Crisis: The Journal of Crisis Intervention and Suicide Prevention. (2014) 35:289–91. doi: 10.1027/0227-5910/a000293 25297514

[13] Roberts B Makhashvili N Javakhishvili J Karachevskyy A Kharchenko N Shpiker M. Mental health care utilisation among internally displaced persons in Ukraine: results from a nation-wide survey. Epidemiol Psychiatr Sci. (2019) 28:100–11. doi: 10.1017/S2045796017000385 PMC6998949 28747237

[14] Panagioti M Gooding P Tarrier N. Post-traumatic stress disorder and suicidal behavior: A narrative review. Clin Psychol review. (2009) 29:471–82. doi: 10.1016/j.cpr.2009.05.001 19539412

[15] Sabawoon A Keyes KM Karam E Kovess-Masfety V. Associations between traumatic event experiences, psychiatric disorders, and suicidal behavior in the general population of Afghanistan: findings from Afghan National Mental Health Survey. Injury epidemiol. (2022) 9:31. doi: 10.1186/s40621-022-00403-8 PMC9535941 36203184

[16] Morina N Rushiti F Salihu M Ford JD. Psychopathology and well-being in civilian survivors of war seeking treatment: A follow-up study. Clin Psychol Psychother: Int J Theory Pract. (2010) 17:79–86. doi: 10.1002/cpp.v17:2 20041418

[17] Lim ICZ Tam WW Chudzicka-Czupała A McIntyre RS Teopiz KM Ho RC. Prevalence of depression, anxiety and post-traumatic stress in war-and conflict-afflicted areas: A meta-analysis. Front Psychiatry. (2022) 13:978703. doi: 10.3389/fpsyt.2022.978703 36186881 PMC9524230

[18] Belete H Misgan E Belete T. Prevalence and associated factors of suicidal behavior among patients and residents in northwest Ethiopia. Front Psychiatry. (2021) 12:560886. doi: 10.3389/fpsyt.2021.560886 34646166 PMC8502868

[19] Tarrier N Gregg L. Suicide risk in civilian PTSD patients: Predictors of suicidal ideation, planning and attempts. Soc Psychiatry Psychiatr epidemiol. (2004) 39:655–61. doi: 10.1007/s00127-004-0799-4 15300376

[20] Ursano RJ Mash HBH Kessler RC Naifeh JA Fullerton CS Aliaga PA. Factors associated with suicide ideation in US Army soldiers during deployment in Afghanistan. JAMA netw Open. (2020) 3:e1919935. doi: 10.1001/jamanetworkopen.2019.19935 31995212 PMC6991281

[21] Shen Y-C Arkes J Williams TV. Effects of Iraq/Afghanistan deployments on major depression and substance use disorder: analysis of active duty personnel in the US military. Am J Public Health. (2012) 102:S80–S7. doi: 10.2105/AJPH.2011.300425 PMC3496458 22390609

[22] Nock MK Millner AJ Joiner TE Gutierrez PM Han G Hwang I. Risk factors for the transition from suicide ideation to suicide attempt: Results from the Army Study to Assess Risk and Resilience in Servicemembers (Army STARRS). J Abnormal Psychol. (2018) 127:139. doi: 10.1037/abn0000317 PMC5851467 29528668

[23] Eskin M Akoglu A Uygur B. Traumatic life events and problem solving skills in psychiatric outpatients: Their relationships with suicidal behavior. Turkish J Psychiatry. (2006) 17:266–75. 17183443

[24] Tinsae T Shumet S Tadesse G Takelle GM Rtbey G Melkam M. Post-traumatic stress disorder in the Ethiopian population dwelling in war-affected communities: a systematic review and meta-analysis. Front Psychiatry. (2024) 15:1399013. doi: 10.3389/fpsyt.2024.1399013 38784164 PMC11112411

[25] Abbink G. The politics of conflict in Northern Ethiopia, 2020-2021: a study of war-making, media bias and policy struggle Vol. 152. Leiden, the Netherlands: African Studies Centre Leiden: The Netherlands, ASCL Working Paper (2021).

[26] Asefa EY Haile AB Mohamed OY Berhanu D. The magnitude of gender-based violence, health consequences, and associated factors among women living in post-war woredas of North Shewa zone, Amhara, Ethiopia, 2022. Front Global women's Health. (2024) 5:1335254. doi: 10.3389/fgwh.2024.1335254 PMC11106405 38774250

[27] Pedersen D. Political violence, ethnic conflict, and contemporary wars: broad implications for health and social well-being. Soc Sci med. (2002) 55:175–90. doi: 10.1016/S0277-9536(01)00261-1 12144134

[28] Bifftu BB Tiruneh BT Dachew BA Guracho YD. Prevalence of suicidal ideation and attempted suicide in the general population of Ethiopia: a systematic review and meta-analysis. Int J Ment Health sys. (2021) 15:1–12. doi: 10.1186/s13033-021-00449-z PMC7992356 33761982

[29] Abdu Z Hajure M Desalegn D. Suicidal behavior and associated factors among students in Mettu University, South West Ethiopia, 2019: An institutional based cross-sectional study. Psychol Res Behav Manage. (2020) 13:233–43. doi: 10.2147/PRBM.S240827 PMC7061437 32184684

[30] Dv S. The Mini-International Neuropsychiatric Interview (MINI): the development and validation of a structured diagnostic psychiatric interview for DSM-IV and ICD-10. J Clin Psychiatry. (1998) 59:22–33. doi: 10.4088/JCP.5640 9881538

[31] Roaldset JO Linaker OM Bjørkly S. Predictive validity of the MINI suicidal scale for self-harm in acute psychiatry: a prospective study of the first year after discharge. Arch suicide Res. (2012) 16:287–302. doi: 10.1080/13811118.2013.722052 23137219

[32] Belete H Misgan E. Suicidal behaviour in postnatal mothers in northwestern Ethiopia: a crosssectional study. BMJ Open. (2019) 9:e027449. doi: 10.1136/bmjopen-2018-027449 PMC6756460 31530587

[33] Ruggiero KJ Ben KD Scotti JR Rabalais AE. Psychometric properties of the PTSD Checklist— Civilian version. J trauma stress. (2003) 16:495–502. doi: 10.1023/A:1025714729117 14584634

[34] Jaranson JM Butcher J Halcon L Johnson DR Robertson C Savik K. Somali and Oromo refugees: correlates of torture and trauma history. Am J Public Health. (2004) 94:591–8. doi: 10.2105/AJPH.94.4.591 PMC1448304 15054011

[35] Blanchard EB Jones-Alexander J Buckley TC Forneris CA. Psychometric properties of the PTSD checklist (PCL). Behav Res Ther. (1996) 34:669–73. doi: 10.1016/0005-7967(96)00033-2 8870294

[36] Wilkins KC Lang AJ Norman SB. Synthesis of the psychometric properties of the PTSD checklist (PCL) military, civilian, and specific versions. Depression anxiety. (2011) 28:596–606. doi: 10.1002/da.20837 21681864 PMC3128669

[37] Asnakew S Shumet S Ginbare W Legas G Haile K. Prevalence of post-traumatic stress disorder and associated factors among Koshe landslide survivors, Addis Ababa, Ethiopia: a community-based, cross-sectional study. BMJ Open. (2019) 9:e028550. doi: 10.1136/bmjopen-2018-028550 PMC6609084 31256034

[38] Gelaye B Williams MA Lemma S Deyessa N Bahretibeb Y Shibre T. Validity of the patient health questionnaire-9 for depression screening and diagnosis in East Africa. Psychiatry Res. (2013) 210:653–61. doi: 10.1016/j.psychres.2013.07.015 PMC3818385 23972787

[39] Kocalevent R-D Berg L Beutel ME Hinz A Zenger M Härter M. Social support in the general population: standardization of the Oslo social support scale (OSSS-3). BMC Psychol. (2018) 6:1–8. doi: 10.1186/s40359-018-0249-9 30016997 PMC6050647

[40] Motrico E Moreno-Küstner B de Dios Luna J Torres-González F King M Nazareth I. Psychometric properties of the List of Threatening Experiences—LTE and its association with psychosocial factors and mental disorders according to different scoring methods. J Affect Disord. (2013) 150:931–40. doi: 10.1016/j.jad.2013.05.017 23726778

[41] Teferi KA. Psychoactive substance abuse and intention to stop among students of Mekelle University, Ethiopia. Mekelle University, Tigray Region, Ethiopia (2011).

[42] Asante KO Quarshie EN-B Onyeaka HK. Epidemiology of suicidal behaviours amongst schoolgoing adolescents in post-conflict Sierra Leone. J Affect Disord. (2021) 295:989–96. doi: 10.1016/j.jad.2021.08.025 34706473

[43] Amare T Meseret Woldeyhannes S Haile K Yeneabat T. Prevalence and associated factors of suicide ideation and attempt among adolescent high school students in Dangila Town, Northwest Ethiopia. Psychiatry J. (2018) 2018:7631453. doi: 10.1155/2018/7631453 29992132 PMC6016154

[44] Ferrada-Noli M Asberg M Ormstad K Lundin T Sundbom E. Suicidal behavior after severe trauma. Part 1: PTSD diagnoses, psychiatric comorbidity, and assessments of suicidal behavior. J Trauma Stress. (1998) 11:103–12. doi: 10.1023/A:1024461216994 9479679

[45] Nesterko Y Haase E Schönfelder A Glaesmer H. Suicidal ideation among recently arrived refugees in Germany. BMC Psychiatry. (2022) 22:183. doi: 10.1186/s12888-022-03844-z 35291976 PMC8922739

[46] Bhardwaj A Bourey C Rai S Adhikari RP Worthman CM Kohrt BA. Interpersonal violence and suicidality among former child soldiers and war-exposed civilian children in Nepal. Global Ment Health. (2018) 5:e9. doi: 10.1017/gmh.2017.31 PMC5827420 29507745

[47] Stanley IH Hom MA Boffa JW Stage DRL Joiner TE. PTSD from a suicide attempt: An empirical investigation among suicide attempt survivors. J Clin Psychol. (2019) 75:1879–95. doi: 10.1002/jclp.v75.10 PMC6744326 31332796

[48] Ferrada‐Noli M Asberg M Ormstad K. Suicidal behavior after severe trauma. Part 2: The association between methods of torture and of suicidal ideation in posttraumatic stress disotrder. J Trauma Stress. (1998) 11:113–24. doi: 10.1023/A:1024413301064 9479680

[49] Alem A Jacobsson L Kebede D Kullgren G. Awareness and attitudes of a rural Ethiopian community toward suicidal behaviour: A key informant study in Butajira, Ethiopia. Acta Psych Scandinavica. (1999) 100:65–9. doi: 10.1111/j.1600-0447.1999.tb10696.x 10470357

[50] Fekadu A Medhin G Selamu M Shiferaw T Hailemariam M Rathod SD. Non-fatal suicidal behaviour in rural Ethiopia: a cross-sectional facility-and population-based study. BMC Psychiatry. (2016) 16:1–9. doi: 10.1186/s12888-016-0784-y 27000122 PMC4802839

[51] Eskin M Baydar N Harlak H Hamdan M Mechri A Isayeva U. Cultural and interpersonal risk factors for suicide ideation and suicide attempts among Muslim college students from 11 nations. J Affect Disord. (2021) 294:366–74. doi: 10.1016/j.jad.2021.07.050 34315098

[52] Chan LF Thambu M. Cultural factors in suicide prevention. In: The international handbook of suicide prevention. Oxford, UK: Wiley-Blackwell (2016). p. 541–55.

[53] Ovuga E Boardman J Wassermann D. Prevalence of suicide ideation in two districts of Uganda. Arch Suicide Res. (2005) 9:321–32. doi: 10.1080/13811110500182018 16179328

[54] Wagenaar BH Hagaman AK Kaiser BN McLean KE Kohrt BA. Depression, suicidal ideation, and associated factors: a cross-sectional study in rural Haiti. BMC Psychiatry. (2012) 12:1–13. doi: 10.1186/1471-244X-12-149 22992379 PMC3515455

[55] Kinyanda E Kizza R Levin J Ndyanabangi S Abbo C. Adolescent suicidality as seen in rural northeastern Uganda. Crisis. (2011) 3. doi: 10.1027/0227-5910/a000059 21371970

[56] Puyat JH Gastardo-Conaco MC Natividad J Banal MA. Depressive symptoms among young adults in the Philippines: Results from a nationwide cross-sectional survey. J Affect Disord Reports. (2021) 3:100073. doi: 10.1016/j.jadr.2020.100073

[57] Nurtanti S Handayani S Ratnasari NY Husna PH Susanto T. Characteristics, causality, and suicidal behavior: a qualitative study of family members with suicide history in Wonogiri, Indonesia. Front Nursing. (2020) 7:169–78. doi: 10.2478/fon-2020-0016

[58] Pitman A Osborn D King M Erlangsen A. Effects of suicide bereavement on mental health and suicide risk. Lancet Psychiatry. (2014) 1:86–94. doi: 10.1016/S2215-0366(14)70224-X 26360405

[59] Sassia S. Long-term unemployment and its influence on the emergence of suicidal thoughts. Tobacco Regul Sci (TRS). (2023), 5270–89.

[60] Markovic M. Mental health consequences of war and post-conflict development: A case study on Bosnia and Herzegovina. (2009).

[61] Mann J Currier D. Stress, genetics and epigenetic effects on the neurobiology of suicidal behavior and depression. Eur Psychiatry. (2010) 25:268–71. doi: 10.1016/j.eurpsy.2010.01.009 PMC2896004 20451357

[62] Krysinska K Lester D. Post-traumatic stress disorder and suicide risk: a systematic review. Arch suicide Res. (2010) 14:1–23. doi: 10.1080/13811110903478997 20112140

[63] Yıldız M. Stressful life events and adolescent suicidality: An investigation of the mediating mechanisms. J Adolescence. (2020) 82:32–40. doi: 10.1016/j.adolescence.2020.05.006 32562924

[64] Wan L-P Yang X-F Liu B-P Zhang Y-Y Liu X-C Jia C-X. Depressive symptoms as a mediator between perceived social support and suicidal ideation among Chinese adolescents. J Affect Disord. (2022) 302:234–40. doi: 10.1016/j.jad.2022.01.061 35090945

[65] Joiner TE. Why people die by suicide. Cambridge, Massachusetts, USA: Harvard University Pres (2005).

[66] Organization WH. Suicide in the world: global health estimates. Geneva, Switzerland: World Health Organization (2019).

